# Synthesis and Characterization of PMBN as A Biocompatible Nanopolymer for Bio-Applications 

**DOI:** 10.22074/cellj.2016.4119

**Published:** 2017-02-22

**Authors:** Puria Motamed Fath, Fatemeh Yazdian, Rogayyeh Jamjah, Bahman Ebrahimi Hosseinzadeh, Maede Rahimnezhad, Razi Sahraeian, Ashrafalsadat Hatamian

**Affiliations:** 1 Faculty of New Sciences and Technologies, University of Tehran, Tehran, Iran; 2 Iran Polymer and Petrochemical Institute, Tehran, Iran

**Keywords:** Nano, Polymer, Drug Delivery System

## Abstract

**Objective:**

Poly [2-methacryloyloxyethyl phosphoryl choline (MPC)-co-n-buthyl methacrylate (BMA)-co-p-nitrophenyl-oxycrabonyl poly ethylene glycol-methacrylate (ME- ONP)] (PMBN), a biocompatible terpolymer, is a unique polymer with applications that range from drug delivery systems (DDS) to scaffolds and biomedical devices. In this research, we have prepared a monomer of p-nitrophenyl-oxycarbonyl poly (ethylene glycol) methacrylate (MEONP) to synthesize this polymer. Next, we designed and prepared a smart, water soluble, amphiphilic PMBN polymer composed of MPC, BMA, and MEONP.

**Materials and Methods:**

In this experimental study, we dissolved MPC (4 mmol, 40% mole fraction), BMA (5 mmol, 50% mole fraction), and MEONP (1 mmol, 10% mole fraction) in 20 ml of dry ethanol in two necked flasks equipped with inlet-outlet gas. The structural characteristics of the synthesized monomer and polymer were determined by Fourier transform infrared spectroscopy (FT-IR), proton nuclear magnetic resonance (H-NMR), dynamic light scattering (DLS), gel permeation chromatography (GPC), scanning electron microscope (SEM), and transmission electron microscope (TEM) analyses for the first time. We treated the polymer with two different cell lines to determine its biocompatibility.

**Results:**

FT-IR and H-NMR analyses confirmed the synthesis of the polymer. The size of polymer was approximately 40 nm with a molecular weight (MW) of 52 kDa, which would be excellent for a nano carrier. Microscopic analyses showed that the polymer was rodshaped. This polymer had no toxicity for individual cells.

**Conclusion:**

We report here, for the first time, the full properties of the PMBN polymer. The approximately 40 nm size with an acceptable zeta potential range of -8.47, PDI of 0.1, and rod-shaped structure indicated adequate parameters of a nanopolymer for nano bioapplications. We used this polymer to design a new smart nano carrier to treat leukemia stem cells based on a target DDS as a type of bio-application.

## Introduction

Recently, biopolymers and biocompatible polymers are a matter of interest because of their specifications. One of the most important parameters is their capability to be used in bio- applications such as drug delivery systems (DDS) and biosensors. Interestingly, their side effects either decrease or disappear. Their use reduces the rejection problem associated with artificial organs or scaffolds (1,3). The 2-methacryloyloxyethyl phosphorylcholine (MPC) polymer has the same polar group (phosphorylcholine) as biomembranes and possesses excellent biocompatibility such as the lack of protein absorption and platelet adhesion, solubility in the liquid phases, stability at a wide range of pH values, and lack of immune reactions (4,6). MPC polymers, because of their biocompatibility properties, are used as surface modifiers for many medical instruments (7,9). The PMB polymer has an MPC unit and an n-butyl methacrylate (BMA) block which, although extremely hydrophilic, can be dissolved in water (10,11). MPC polymers with hydrophobic monomer units such as poly( L-lactide-co-caprolactone-co-glycolide (PLCG)/PMB (12), cholesterol-end-capped poly (MPC) (CMPC) (13), PMBN (14), poly ( MPC-block-DEA ( 2-(N,N-diethylamino) ethyl methacrylate)) (15), poly ( DMAPAA ( N-[3-(dimethylamino)propyl] acrylamide)- co-MPC-co-SA (stearyl acrylate)) (16), and poly ( MPC-co-DAMA ( 2-methyl-acrylic acid 2-[( 2-(dimethylamino)-ethyl-methyl-amino]- ethyl ester) (17) can solubilize hydrophobic drugs or even be used for gene therapy. 

Goda et al. (18), in a review article, mentioned the role of the MPC polymer that has a group of phosphatidylcholines (PC). Since the outer surface of the plasma membrane of eukaryotes contain PC groups, the MPC molecule has been formed to create a physically neutral surface that the dipole structure of the phosphatidylcholine group in the molecule was responsible for neutralization of the biological characteristics of the MPC. Other polymer molecules could be designed easily and efficiently where they could be used in many biomedical applications. One of the most important polymers is PMBN. Polymers made of MAONP monomers that contain active ester groups probably attach to the other biological molecules with a free -NH2 group and can be used for the nano- systems in drug industry or biosensors. The direct diffusion of amphiphilic polymer into the plasma membrane is possible without causing toxicity. This capability is very attractive, since all the macro molecules formed could not pass through the plasma membrane barrier without breaking the petit layers or protective bio systems. Therefore, according to the features mentioned above, MPC polymers (particularly PMBN) have been increasingly used in all areas such as DDS (19), DNA microarray (20), nano biosensors (21), biochips (22), and tissue engineering (23). PMBN consists of three monomers (MPC, BMA, and MENOP) whereas PMB has monomers of MPC and BMA. The MEONP monomer make give the targeting ability to polymer because of active ester groups as was discussed before. 

In the current study, we used the conventional radical polymerization method, initially employed by Konno et al. (21) to synthesize the water soluble, amphiphilic terpolymer, PMBN. Miyata et al. (19) found that when PMBN conjugated to preS1 as a domain surface of the hepatitis B antigen, and loaded by paclitaxel (PTX), showed a substantially enhanced capacity to heal human hepatocyte diseases without any side effects when used in DDS. In another study reported by Shimada et al. (24), PMBN loaded by PTX and conjugated to epidermal growth factor (EGF-PMBN- PTX) could be a novel target system to cure tumors that overexpressed EGF receptors. Also, because of the great specification of this polymer due to its monomers and the polymer itself, it has been used to cover many nano particles in various applications (21,25,28). The PMBN polymer was characterized by different assays to determine its properties and capacity for use in nano bioapplications. Unfortunately, we did not locate any clear characteristics or features that pertain to PMBN as a novel biocompatible polymer. This polymer might possess biocompatibility because of its structure. The MPC monomer consists of phosphoryl choline (PC) groups which simulate cell membrane structure. Hydrophobic drugs, and components could be loaded by the BMA monomer and, conjugation of Bio molecules will be done easily by MEONP units. 

## Materials and Methods

### Materials

MPC, BMA, p-nitrophenyl chloroformate, triethylamine (TEA), 2, 2’-Azobis (2-methylpropionitrile) (AIBN), diethyl ether, chloroform, the MTT kit and ethanol were purchased from Sigma Aldrich Co., Germany without further purification. The poly (ethylene glycol) monomethacrylate (Blenmer PE-200) was prepared from Polysciences Co., USA. The Kg-1 and Molt4 cell lines were purchased from the Cell Line Bank at Pasteur Institute, Iran. 

### Experiments

#### Monomer synthesis (MEONP)

In this experimental study, TEA (8.1 g), PE- 200 (22.0 g), and chloroform (50 ml) were poured into a flask that contained a magnet and dropping dropper funnel. Then, the flask was placed in a bath at a temperature of -30˚C which was kept constant with nitrogen and acetonitrile. Subsequently, p-nitrophenyl chloroformate (16.2 g) was dissolved in chloroform (40 ml) and poured to dropper funnel. The solution was added to the flask during 1hour at -30˚C very slowly when was blending by magnetic stirrer. The flask was kept at this temperature for 2 hours and the solution was thoroughly mixed. Later, the formed deposits (TEAC) were filtered and a final solution was recovered by a Rotary evaporator. Dry diethyl ether was added to the residue and filtered. Finally, the solution was allowed to evaporate to obtain a pure yellow oily liquid (21). The structure of synthesized MEONP was confirmed by Fourier transform infrared spectroscopy (FT-IR, FT-IR MB102, BOMEM Inc., Switzerland) using the KBr pellet technique. Also, proton nuclear magnetic resonance (H-NMR) instrument (H-NMR DRX 300, Bruker Avance Co., USA) applied by CDCI3 as the solvents and tetramethylsilane (TMS) as the internal standard. 

#### Synthesis of a water soluble, amphiphilic polymer (PMBN)

MPC (4 mmol), BMA (5 mmol), and MEONP (1 mmol) were dissolved in 20 ml of dry ethanol and poured into a two-necked flask equipped with a gas inlet-outlet. Then, AIBN (0.1 mmol) was added as an initiator. First, N_2_ gas was blown for 10 minutes into the mixture and the flask. Next, the flask was sealed and placed in an oil bath at 60˚C . The solution was thoroughly blended at that temperature for 4 hours under a N_2_ gas atmosphere. Finally, the mixture was added to the cold diethyl ether solvent to obtain a polymer as a white deposit. We purified this product by dissolving it in a minimum volume of pure ethanol and some cold diethyl ether. The final product was placed in a vacuum oven for 3 days at 50˚C until the product dried and became crystallized. The process and volumes have certain differences compared with the work carried out by Konno et al. (21). If the synthesis was done per their procedure, the desired polymer would not be achieved. For confirmation of its structure, we conducted FT-IR and H-NMR measurements as previously mentioned. Dynamic light scattering (DLS, DLS Nano-ZS, Malvern Co., UK) was performed to calculate the size of the nanoparticles, size distribution, and Zeta potential of the polymer. We used the gel permeation chromatography (GPC) technique in a water/methanol (3:7) mixture as a solvent with poly (ethylene glycol) as the standard sample to determine the molecular weight (MW) of PMBN. Finally, the shape and size of synthesized polymer were measured by scanning electron microscope (SEM, SEM VEGA II, Tescan Co., Czech Republic) and transmission electron microscope (TEM, TEM EM10C-80 KV, Zeiss Co., Germany). 

### Toxicity analysis

The 3-(4, 5-dimethylthiazol-2-yl)-2, 5-diphenyl tetrazolium bromide or MTT assays were carried out to measure the cytotoxicity of the polymer. Briefly, the two different cell lines (Kg-1 and Molt 4) were grown on 96-well plates and incubated overnight at 37˚C . The initial cell number for each well was 105 cells. The Kg-1 cell lines is a type of acute myeloid leukemia (AML) cell line that over expresses the CD123 receptor on its surface. This overexpression property makes this cell line as a good choice for the further studies all the authors. The negative control, Molt 4 is an acute lymphoblastic leukemia (ALL) cell line that does not express CD123. The desired concentration of PMBN (10-1200 nM) was added to the plates. After 72 hours, we rinsed the cells with PBS and performed the MTT assay according to the kit’s protocol. The absorbance of each well was measured by a microplate reader (Epoch, Biotek Co., USA) using a 570 nm test wavelength and 630 nm as a reference wavelength. 

### Statistical analysis

All data were analyzed by the Mann-Whitney U test. Data were considered significant at P˂0.05. All tests and experiments were performed in triplicate and repeated at least three independent times. 

## Results

### MEONP characterization 

**Fig.1:**
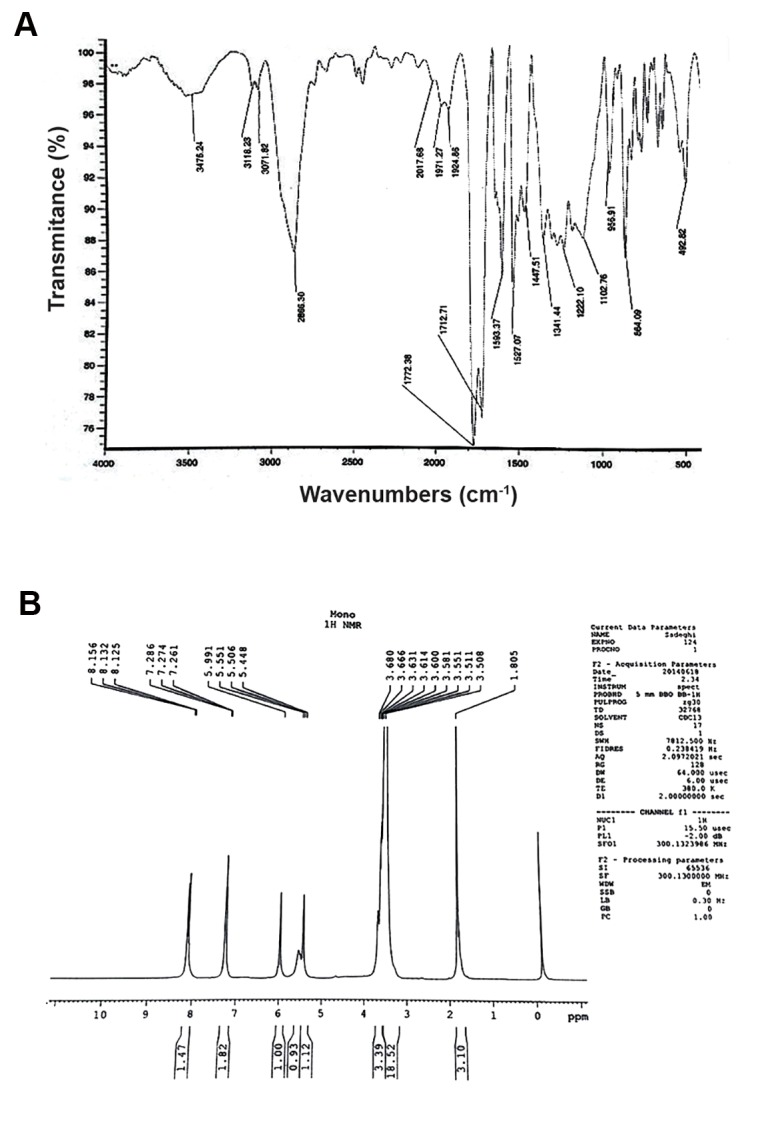
The MEONP monomer was analyzed by Fourier transform infrared spectroscopy and proton nuclear magnetic resonance to confirm its structure. A. FT-IR graph of the synthesized MEONP monomer that demonstrated its structure and recognizable peaks and B. Spectrum of H-NMR measurement of synthesized MEONP which showed specific peaks at 1.8, 3.6, 5.5, 6.0, 7.2 and 8.1 ppm.

The structure of synthesized monomer, MEONP, was accepted and confirmed by FT-IR and H-NMR. The predictable peaks are shown Figure 1. 

### PMBN characterization by different methods

**Fig.2:**
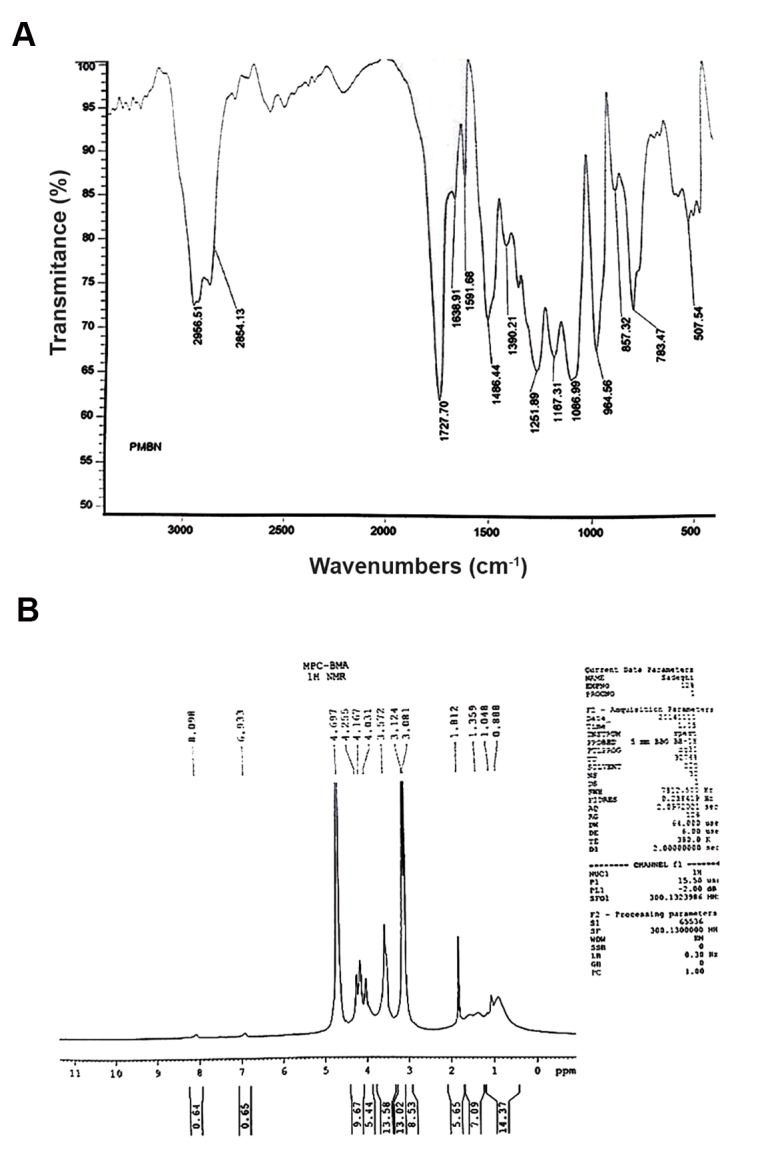
The MEONP monomer was analyzed by Fourier transform infrared spectroscopy and proton nuclear magnetic resonance to confirm its structure. A. FT-IR graph of synthesized PMBN polymer with desired peaks is shown and B. H-NMR spectrum of PMBN with specific peaks at 1.81, 4-4.3, 3.5 1.0, 1.3, 7.0 and 8.1 ppm.

**Fig.3:**
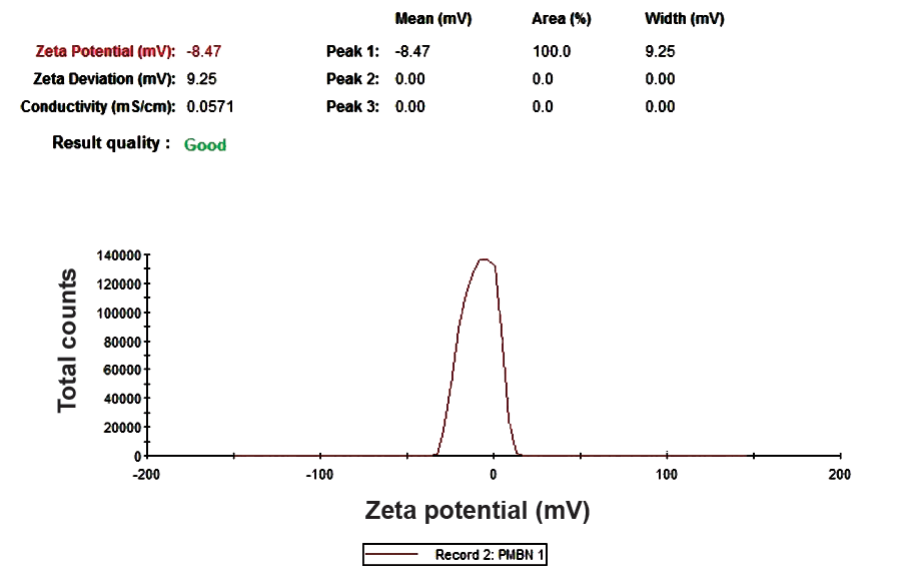
Zeta potential of polymer was analyzed by dynamic light scattering to found out it was cation or anion. PMBN with -8.47 mV zeta potential is an anionic particle.

After synthesize of the PMBN polymer, we used FT-IR (Fig .2A) and H-NMR (Fig .2B) measurements to confirm the structure. The achieved peaks were expected according to its monomers. The next measurement was the size and zeta potential analysis by the DLS instrument (Fig .3) to determine whether this polymer was cationic or anionic. 

**Fig.4:**
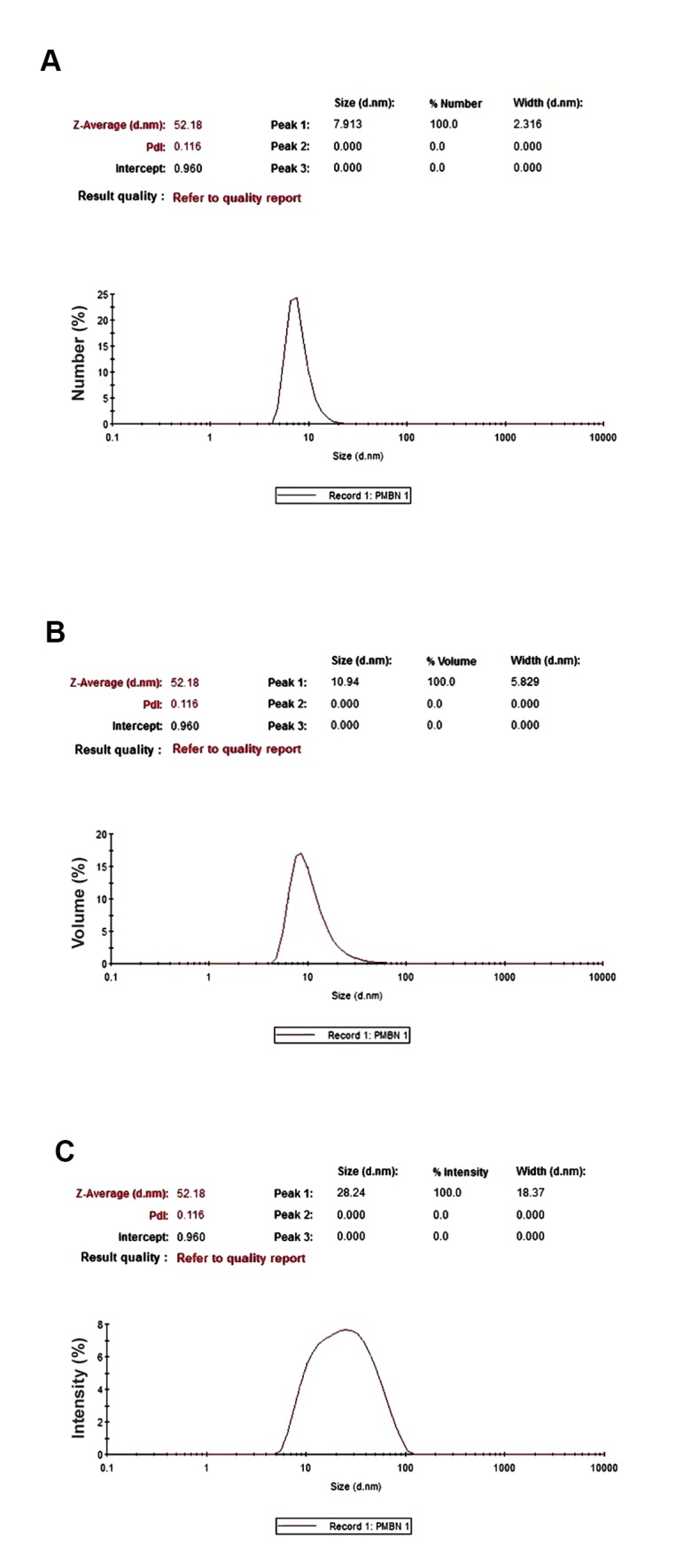
Size of polymer was analyzed by dynamic light scattering (DLS) to figured out it was had nano structure or not. A. Measurement based on number index that showed a mean diameter of 52.18 nm, B. Measurement based on volume index that indicated a mean diameter of 52.18 nm, and C. Measurement based on intensity index that determined the polymer size at 52.18 nm. The poly dispersity index (PDI) of PMBN was 0.116.

Figure 4 shows the size calculation of the polymer based on number (a), volume (b), and intensity (c) indices of DLS analysis. If the indices showed a closed number, it could be concluded that the polymer was synthesized well according to the visualized applications. 

**Fig.5:**
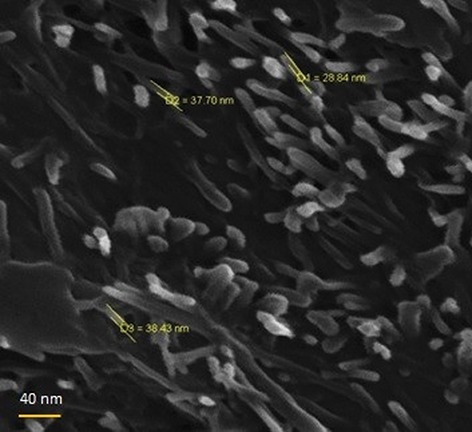
Scanning electron microscope (SEM) image of synthesized PMBN polymer with magnification of 70.00 kx which shows size ~35 nm and confirmed the rod shaped structure of the polymer.

**Fig.6:**
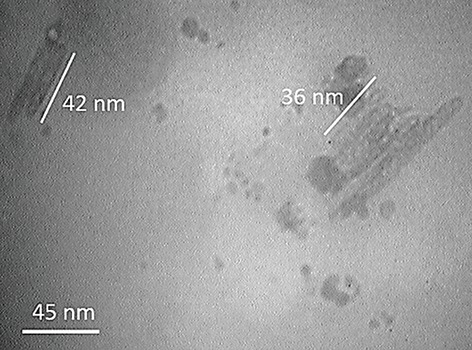
Transmission electron microscope (TEM) images of the synthesized PMBN polymer with a magnification of 100 kx. The rod shaped structure of the PMBN polymer with a size of approximately 40 nm is confirmed.

In order to determine the MW of this polymer, we conducted GPC assays by using a hydrophilic column and determined that the MW of PMBN was 52000 Da. We performed SEM imaging to determine the size and shape of synthesized polymer (Fig .5). The results indicated that the polymer had a nano structure and confirmed the results of the DLS measurement. The last experiment, TEM imaging, confirmed the polymer’s structure, shape and size (Fig .6). The analysis was carried out by a formvar carbon coated grid Cu Mesh 300 for all samples. In this study, authors are working on conjugation of polymer with IL-3 as the ligand to make a smart nano carrier for leukemia therapy. The conjugation results indicated that 76% of the available sites of the polymer linked to IL-3 according to equation 1. 

$$\frac{\left(A_{a}-A_{b}\right)}{A_{c}}x100=\frac{1.9-0.3}{2.1}x100=77.2\%$$

A_a_; Absorbance of PMBN hydrolysis in water, A_b_; Absorbance of PMBN-IL3 hydrolysis in water, and A_c_; Absorbance of PMBN hydrolysis in NaOH as the reference absorbance.

Thereafter, PTX (hydrophobic drug) and 90% of the obtainable positions of the PMBN polymer were loaded which was calculated by equation 2. 

$$\frac{\left(A_{a}-A_{b}\right)}{A_{a}}x100=\frac{1.92-0.02}{1.92}x100=89.6\%$$

A_a_; Absorbance of PTX in solution before loading and A_b_; Absorbance of PTX in the solution after loading.

Synthesis and Characterization of PMBN as A Biocompatible Nanopolymer

### MTT assay

**Fig.7:**
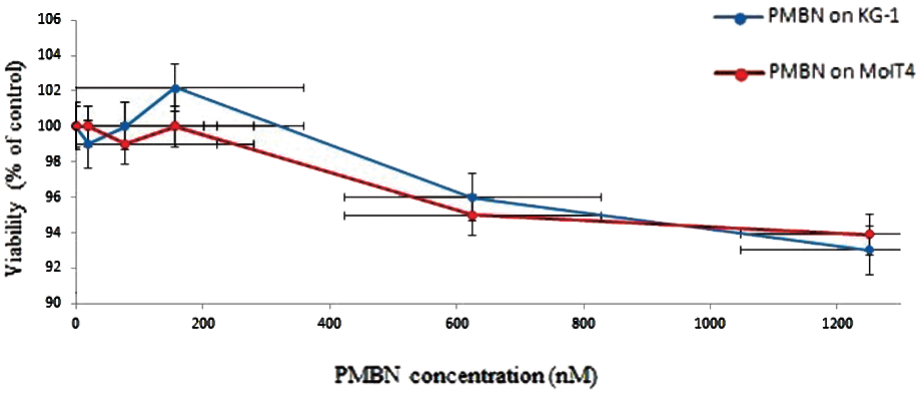
MTT assay of the PMBN polymer on two different cell lines, KG-1 and Molt 4, to discover toxicity behavior of the synthesized polymer. At higher concentrations of this polymer, about 1200 nM, the viability reduction is less than 5%.

We conducted the MTT assay to determine if the polymer had any toxicity for cells individually. The data indicated no toxicity, as was expected (Fig .7), even at high concentrations of the polymer. 

## Discussion

We confirmed the structure of the synthesized monomer, MEONP, by measurements from FT- IR and H-NMR. The recognizable peaks of FT- IR analysis at 1712 cm^-1^ is an ester carbonyl group, whereas the 1772 cm^-1^ peak indicates a carbonate group. The 2860 cm^-1^ peak is for an aliphatic bond and 3100 cm^-1^ represents an aromatic C-H bond. In addition, H-NMR measurements indicated that the peaks at 1.8 and 3.6 ppm were for protons of the methyl connection of an alkane dual bond (CH_3_-C=C) and protons of the poly ethylene glycol portion (OCH_2_CH_2_O), respectively. Alkene protons (H_2_C=C) were shown at 5.5 and 6.0 ppm and the 7.2 and 8.1 ppm values were specific for protons of the aromatic ring. 

After synthesis of the PMBN polymer, we obtained FT-IR and H-NMR measurements for to confirm the structure. The FT-IR graph of PMBN showed specific peaks at 1727 cm^-1^ (carbonyl), 1086 cm^-1^ (POCH_2_), and 964 cm^-1^ [N^+^ (CH_3_)]. Acceptable peaks of H-NMR analysis for PMBN were observed. Both measurements have confirmed that PMBN was synthesized correctly with desired monomers and blocks. Of interest, if the mole fraction of the monomer were to change (e.g., if the ratio rate of the MPC monomer had decreased from 40 to 35%), the PMBN polymer would no longer be water soluble. If the volume of ethanol during synthesis of the polymer decreased to 1/2, the polymer structure would change to a gum, viscous form with remarkably different properties. 

Next, we used DLS analysis to measure size and zeta potential. Zeta potential of the PMBN polymer as a nanoparticle was -8.47 mV which indicates that this nano particle is anionic. Anionic polymers and nano structures such as PMBN have been investigated by other researchers where the results confirmed higher cellular uptake, lower protein adsorption (29), and higher stability under *in vivo* conditions (30,31) compared to cationic polymers. Only anionic nano structures have been FDA approved for treatment application (31). The size calculation of the polymer based on number, volume, and intensity indices of DLS analysis was carried out. The measurements of polymer based on these three indices has confirmed the 52.18 nm size of the particle. These scales are ideal for a nano polymer. The intensity based measurement graph was not as well-defined as the two other graphs because it derived from coagulated particles formed in the sample with very low volume which would not be effective for number and volume analyses. The PMBN Poly Dispersity Index is 0.116. This is an acceptable index to be used for a nano structure, even for DDS. Poly dispersity indices equal or less than 0.1 are typically referred to as "mono dispersive". 

We conducted GPC assays with a hydrophilic column in order to determine the MW of the polymer. The MW of PMBN was 52000 Da, an acceptable weight for a nano polymer to be used in bio-applications. In the further study PMBN conjugation of IL-3 with a MW of 15000 Da as a ligand was used for targeting stem cells. After conjugation, the product purification was easily performed from the free ester group (p-nitrophenol) of the MEONP monomer and IL-3 by filtered falcons with a 30000 cut-off. 

SEM was used to determine the size and shape of the synthesized polymer. The scale of the polymer approximated 35 nm, which was close to the results achieved by DLS measurements (52.18 nm). The slight difference between these two analyses was probably due to the hydrodynamic diameter, which was typically larger than diameters determined by SEM and TEM and a function of the capping agent. The synthesized polymer had a rod-shaped structure, which was interesting. This shape indicated that PMBN, as a Terpolymer with hydrophilic and hydrophobic blocks, would easily solubilize hydrophobic components or could be used as an acceptable scaffold for tissue engineering. This structure cleared that instead of MPC polymers which convert to liposomal structure spontaneously, rod shaped structure of polymer with open monomer blocks will be effortlessly active for their functions. Kolher et al. (32) reported that contrary to spherical nanoparticles, rod-shaped nanoparticles (nano rods) appeared to more effectively adhere to the surface of endothelial cells and exhibit increased endothelial specificity *in vitro* as well as *in vivo*. Rod shaped nanoparticles exhibit higher cellular internalization (33) and such particles have be seen to exhibit excellent targeting in tumor xenografts (34). The shape of this nanoparticle is ideal for targeting DDS to be used for specific cell or tissue types. 

In the case of being satisfied with polymer structure, about its shape and size, the last experiment, TEM imaging was carried out. We performed the analysis with formvar carbon coated on a 300 Mesh Cu grid for all samples. The structure is clearly Rod shaped with a size of approximately 40 nm; acceptable results were achieved by SEM and DLS measurements. The synthesized polymer, PMBN, was treated on two types of cell lines, Kg-1 and MolT4. We conducted the MTT assay to discover the toxicity of the polymer on these cells. If the viability of each well was more than 80%, that agent might not have a toxicity effect on cell proliferation. We observed no toxicity or reduction in cell proliferation for both cell lines in the presence of this polymer, even at high concentrations (1200 nM). This data showed the biocompatibility of this polymer and its ability to be used in bio-applications such as targeted DDS or scaffolds. 

## Conclusion

We synthesized a water soluble, amphiphilic polymer, PMBN and characterized its structure and properties for the first time. This polymer with a nano structure size around 30-45 nm, acceptable zeta potential range, PDI index, and MW of 52000 Da is an unlimited, ideal biocompatible polymer for nano bio- applications as previously mentioned. In this study, the targeting of this polymer for AML leukemia stem cell cancer (LSC) therapy is under investigation. The data indicated that the PMBN polymer would be a good choice for bioapplications such as targeted drug delivery or gene therapy. 
